# Normal liver enzymes are correlated with severity of metabolic syndrome in a large population based cohort

**DOI:** 10.1038/srep13058

**Published:** 2015-08-13

**Authors:** Julia Kälsch, Lars P. Bechmann, Dominik Heider, Jan Best, Paul Manka, Hagen Kälsch, Jan-Peter Sowa, Susanne Moebus, Uta Slomiany, Karl-Heinz Jöckel, Raimund Erbel, Guido Gerken, Ali Canbay

**Affiliations:** 1 Department of Gastroenterology and Hepatology, University Hospital, University Duisburg-Essen; 2 Department of Bioinformatics, Straubing Center of Science, University of Applied Science Weihenstephan-Triesdorf; 3 Department of Cardiology, West-German Heart Center, University Hospital, University Duisburg-Essen; 4 Institute of Medical Informatics, Biometry and Epidemiology, University Hospital, University Duisburg-Essen; 5 Regeneration and Repair Group, The Institute of Hepatology, Foundation for Liver Research, London, UK

## Abstract

Key features of the metabolic syndrome are insulin resistance and diabetes. The liver as central metabolic organ is not only affected by the metabolic syndrome as non-alcoholic fatty liver disease (NAFLD), but may contribute to insulin resistance and metabolic alterations. We aimed to identify potential associations between liver injury markers and diabetes in the population-based Heinz Nixdorf RECALL Study. Demographic and laboratory data were analyzed in participants (n = 4814, age 45 to 75y). ALT and AST values were significantly higher in males than in females. Mean BMI was 27.9 kg/m^2^ and type-2-diabetes (known and unkown) was present in 656 participants (13.7%). Adiponectin and vitamin D both correlated inversely with BMI. ALT, AST, and GGT correlated with BMI, CRP and HbA1c and inversely correlated with adiponectin levels. Logistic regression models using HbA1c and adiponectin or HbA1c and BMI were able to predict diabetes with high accuracy. Transaminase levels within normal ranges were closely associated with the BMI and diabetes risk. Transaminase levels and adiponectin were inversely associated. Re-assessment of current normal range limits should be considered, to provide a more exact indicator for chronic metabolic liver injury, in particular to reflect the situation in diabetic or obese individuals.

The liver as central organ for glucose and lipid metabolism is strongly affected by the metabolic syndrome. Thus, non-alcoholic fatty liver disease (NAFLD) represents the hepatologic consequence of Western lifestyles. NAFLD is the most common chronic liver disease in industrialized nations with a prevalence of up to 30% and probably the most common cause of elevated liver enzymes^1^,^2^. According to the National Health and Nutrition Examination Survey, the prevalence of major causes of chronic liver diseases remained stable from the years 1988 to 2008 except for NAFLD. Incidence of NAFLD increased steadily during this time, contributing to the burden of chronic liver disease in the United States^3^. NAFLD is associated with obesity as well as diabetes and could be not only a result of insulin resistance (IR) and metabolic syndrome but rather a major contributor to systemic IR^4^,^5^. NAFLD ranges from simple hepatic steatosis (NAFL) to non-alcoholic steatohepatitis (NASH) with hepatocyte ballooning and inflammatory components. Simple hepatic steatosis generally has a good prognosis, however NASH can lead to cirrhosis and hepatocellular carcinoma (HCC) in up to 15% of patients^6^. Metabolic injury to the liver will probably constitute a major burden for health care systems worldwide in the near future.

By now the metabolic syndrome is a common term and high cholesterol, high blood pressure, or obesity raise the attention of internists regarding the risk for cardiovascular or metabolic complications. Though, despite the very obvious link of NAFLD and metabolic syndrome, the awareness of metabolic liver injury and its connection to cardiovascular risk remains low. Several studies have shown, that normal transaminase levels do neither exclude NAFLD (or NASH) nor progression to advanced fibrosis^7^,^8^. However, aminotransferases are regarded as the main alarm signal for liver diseases or injury before enrolling further diagnostic. Previous studies already discuss the idea to lower normal values and to take metabolic factors into account, especially body mass index (BMI) and sex, which have a significant effect on ALT values^9^,^10^,^11^. Since the origin of obesity may be based in early childhood, hepatologists already claim to revise the values in pediatrics^11^. Still the classic liver serum parameters (ALT, AST, GGT), elevated in most chronic and acute liver diseases, may not be ideal markers for liver injury in NAFLD.

Liver function is crucial for glucose- and fatty acid metabolism and vice versa^12^. Enzymes and signaling pathways involved in hepatic glucose homeostasis contribute to insulin sensitivity. Reciprocally peripheral IR and lipolysis contribute to hepatic steatosis^13^,^14^. Other factors known to contribute to systemic IR and to development of NAFLD are adipokines such as adiponectin. Low adiponectin is associated with obesity, IR, and severity of NAFLD^15^,^16^,^17^. Vitamin D (VD) is also discussed to play an important role in the pathophysiology of IR and VD serum concentrations above 25 ng/ml were associated with a lower risk of type 2 diabetes^18^.

In the present study we aimed to investigate in a large population based cohort, whether serum transaminase levels within normal ranges are associated with metabolic risk and prevalence of diabetes. Associations of adiponectin, systemic inflammation, and VD levels with transaminases and diabetes were analyzed. Previously we were able to generate effective classification models by machine learning techniques^19^,^20^,^21^. Thus, we also aimed to build a model for diabetes prediction from non-invasive parameters.

## Results

### Demographic data

**Figure 1:**
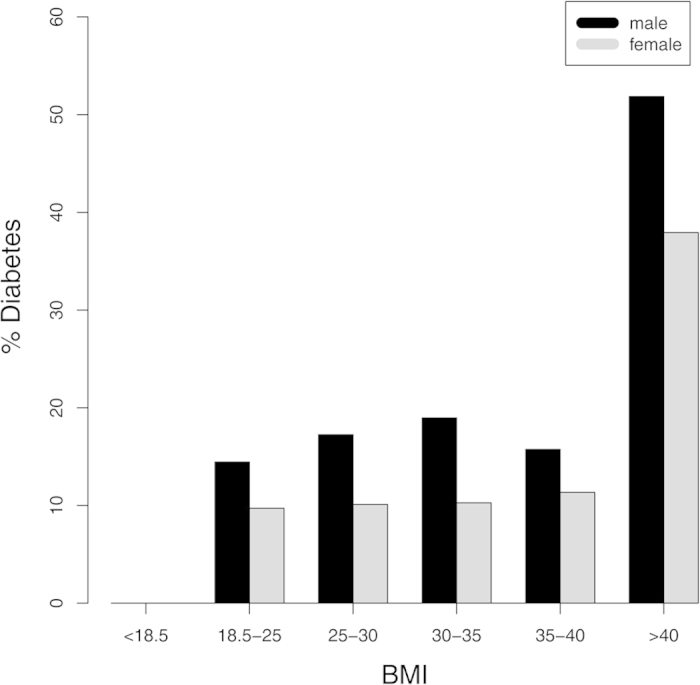
Prevalence of diabetes in the Heinz Nixdorf Recall study by BMI groups. Diabetes was present in below 20% of males and below 10% in females of all BMI groups up to 40 kg/m^2^. In the highest BMI group (obesity grade III; >40 kg/m^2^) more than 50% male subjects and almost 40% female subjects had overt diabetes.

The initial cohort included 4814 participants (2419 female), aged 45 to 75 years (female 59.6 ± 7.8y, male 59.7 ± 7.8y). Detailed demographics are given in Table 1. Men exhibited significantly higher weight (86.2 ± 13.2 kg; *p* < 0.0001) and waist circumference (100.3 ± 10.8 cm; *p* < 0.0001) than women. The mean BMI of the cohort was 27.9 kg/m^2^, suggesting large parts of this population to be overweight or obese. Both women and men were considered overweight by BMI, with men reaching higher values (m: 28.2 ± 4.0 kg/m^2^ *vs.* w: 27.7 ± 5.2 kg/m^2^; *p* < 0.0001). Type 2 diabetes was present in 656 individuals of the HNR cohort (13.7%; Fig. 1). Thereof 397 (8.2%) had previously known type 2 diabetes and 259 (5.4%) had unknown diabetes. Type 2 diabetes was more common in men with 418 (17.5%) male subjects affected compared to 238 female subjects (9.8%). The highest proportion of diabetes was found in subjects with BMI above 40 (approx. 45%).

### Transaminase levels in the HNR Study Cohort were within normal limits, but gradually increase with BMI

**Figure 2:**
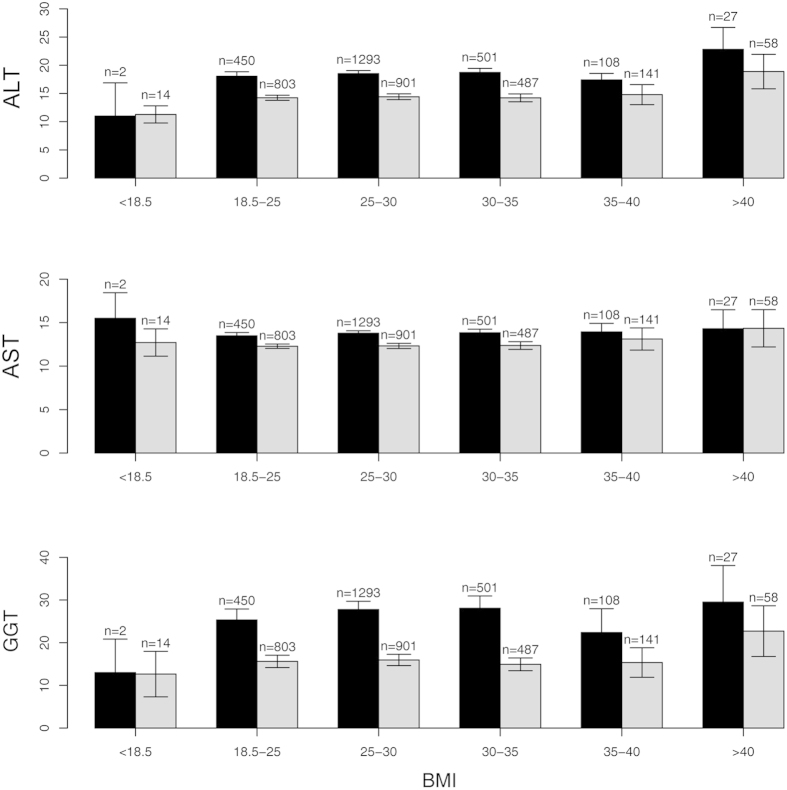
Distribution of classic serum liver parameters in the Heinz Nixdorf Recall study by BMI groups. Alanine aminotransferase (ALT/GPT) and gamma Glutamyltransferase (GGT) exhibited a trend of increased concentrations with higher BMI. Aspartate aminotransferase (AST/GOT) showed no differences between the BMI groups.

Mean ALT (16 ± 8.8 U/l) and mean AST (13 ± 4.6 U/l) remained both well below the common threshold for normal values (<50 U/l for males; <35 U/l for females). ALT was significantly higher in male subjects than in females (19 ± 9.7 U/l vs. 14 ± 7.1 U/l; *p* < 0.0001), which was also observable for AST (14 ± 4.8 U/l vs. 12 ± 4.2 U/l; *p* < 0.0001; Fig. 2).

Subjects were grouped by BMI according to common ranges for underweight, normal weight, overweight, and obesity grades I-III. Serum concentrations of ALT and GGT tended to be higher in higher BMI ranges, though this trend did not reach significance (Fig. 2). Moreover, even in the highest BMI group (>40 kg/m^2^) the mean concentrations were still within normal ranges. A comparison of demographic and metabolic data of individuals with ALT in normal ranges and those with elevated ALT is given in Supplementary Table 1. AST levels were similar in all BMI groups.

### Adiponectin and vitamin D levels are inversely correlated with BMI

**Figure 3:**
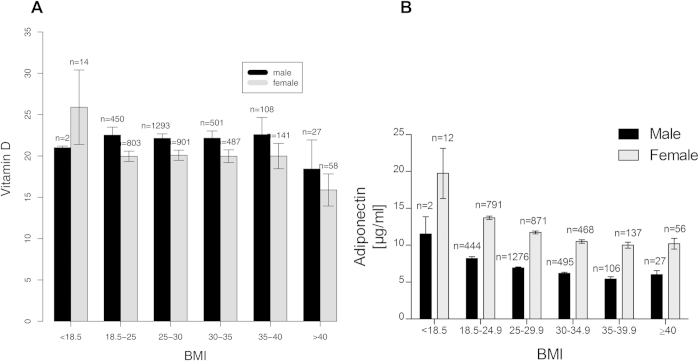
Distribution of metabolic serum markers in the Heinz Nixdorf Recall study by BMI groups. (**A**) Vitamin D serum levels were close to the threshold to insufficiency (20 ng/ml) in all BMI groups <40 kg/m^2^. In the highest BMI group (>40 kg/m^2^) the lowest vitamin D values were observed, with a mean concentration indicating insufficiency. (**B**) Adiponectin was distributed in a similar way as vitamin D with lowest serum concentrations found in the highest BMI group.

For all BMI groups VD was around the threshold for a deficit. Only in the highest BMI category (above 40 kg/m^2^) the mean VD values indicated a true deficit (<20 ng/ml; Fig. 3A). Slightly higher VD levels were found in males compared to females. Serum adiponectin, an adipocytokine with known hepatoprotective and insulin sensitizing properties^22^,^23^,^24^, was low (<15 μg/ml) in all BMI groups. The lowest concentrations were again observed in the highest BMI group (Fig. 3B). Female subjects exhibited higher adiponectin levels than males. While no significant differences between BMI groups were observed for adiponectin and VD, both parameters correlated inversely with BMI (Table 2).

### Transaminase levels correlate with BMI and HbA1c, while adiponectin was inversely correlated with transaminase levels

ALT, AST, and GGT correlated positively with BMI (Table 2). To link transaminase levels with a surrogate parameter of IR, HbA1c was quantified in this cohort. ALT, AST, and GGT significantly correlated with HbA1c. In contrast, adiponectin was inversely correlated with AST, ALT, and GGT. Interestingly, GGT was significantly associated with CRP, a marker of systemic inflammation and associated with obesity, linking hepatocellular injury to systemic inflammation.

### HbA1c, adiponectin, and BMI are efficient predictors of diabetes in the HNR study cohort

**Figure 4:**
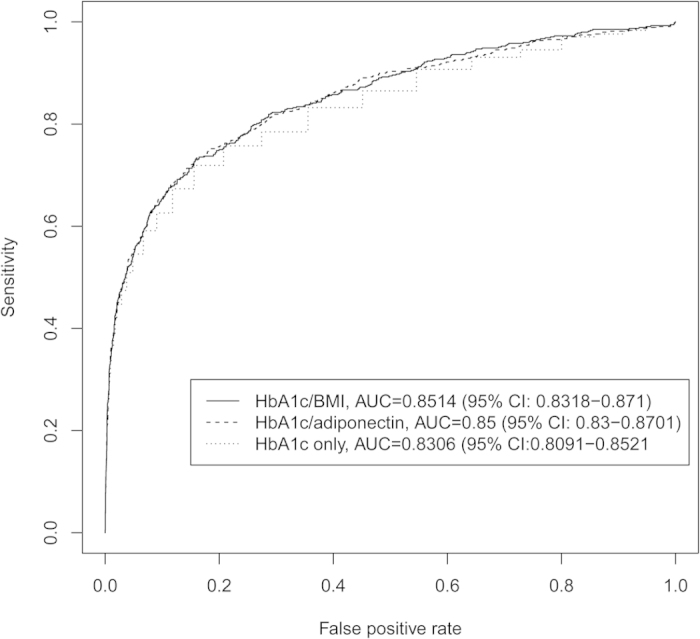
ROC curves of the logistic regression models generated from non-invasive markers. The models generated from serum markers and BMI (see table 3 for list) were able to predict diabetes with a high accuracy.

Utilizing machine learning techniques a computational model was built to identify the most important parameters for prediction of diabetes from the available serum parameters. The model identified HbA1c, adiponectin, and BMI as highly important for the prediction of diabetes (Table 3). These were followed by GGT, vitamin D, and transaminases. Logistic regression models were built, using HbA1c, adiponectin, and/or BMI to predict diabetes from the presented cohort. The model using all three parameters (HbA1c, adiponectin and BMI) as well as the reduced models reached AUC values of 0.85. A model using only HbA1c reached an AUC of 0.83. The ROC curves of the models HbA1c and BMI, HbA1c and adiponectin and HbA1c only are shown in Fig. 4. Comparing the AUC values by the method of De Long *et al.*^25^, it turned out that both, the model of HbA1c + BMI and HbA1c + adiponectin have significant higher AUC values compared to the model that uses only HbA1c (p < 0.001). The differences between the AUC values of the models HbA1c + BMI and HbA1c + adiponectin were not significant (p = 0.7044). By comparing the models at FPRs of 0.1, 0.05, and 0.001 using McNemar’s tests, both, the model using HbA1c + adiponectin as well as the model using HbA1c + BMI showed significant differences at FPR = 0.05 (p < 0.001 and p = 0.002, respectively) compared to the model using only HbA1c. However, only the model using HbA1c and adiponectin showed significant differences for very low FPRs (FPR = 0.01, p = 0.005). A model using HbA1c, adiponectin and BMI did not significantly improve the overall performance compared to the models that use only two parameters. Adding the parameters GGT and vitamin D did not significantly improve the models further (not shown).

## Discussion

Metabolic syndrome is alongside the obesity epidemic of industrialized countries increasingly common. It is hardly surprising that in parallel NAFLD has become the most predominant chronic liver disease in Europe^26^ with up to 75% of NAFLD patients suffering from diabetes^27^. Mild elevations of aminotransferases are a common finding in NAFLD^28^, though in over 25% of patients with advanced NAFLD and NASH, transaminase levels remain within normal levels^7^. In the presented cohort a very low proportion of transaminase levels above normal limits were detected (<2% for ALT). Though, with increasing BMI a parallel increase in ALT and GGT levels was observed. Mean BMI of the cohort suggests a substantial proportion of the population is overweight or obese. Moreover, overt type 2 diabetes was found in roughly 14%, while current German cross-sectional studies indicate a prevalence of approximately 7.2% in the general population^29^,^30^, though in these cases only known diabetes was taken into account. This data suggests that a relevant proportion of the analyzed collective may have undiagnosed metabolic syndrome. These individuals are at a greater risk to develop NASH and consecutively cirrhosis or HCC^31^. The main indicator to enroll further diagnostics for liver injury in clinician’s daily routine are liver transaminases. Thus, current normal values might miss a significant amount of individuals already developing chronic metabolic liver disease and presenting with transaminase values in the upper normal levels^32^,^33^.

Despite mostly marginal elevations in patients with overweight and obesity, serum liver enzymes have previously been associated to metabolic syndrome or its components. In the Cyprus Metabolism study, Liu *et al.* demonstrated a clear association between ALT and GGT with metabolic risk factors, including IR^34^. Similar associations between transaminase levels and diabetes have been shown, however, data is still scarce^35^. ALT, AST, and GGT in the presented cohort were each correlated with HbA1c levels and BMI. Additionally, GGT was associated with CRP, suggesting a connection to systemic inflammation beyond metabolic associations. This may support previous suggestion of GGT as biomarker for atherosclerosis^36^. Although classic serum markers for liver damage were not elevated above normal ranges, higher values may still indicate metabolic alterations or even injury by IR and diabetes.

Apart from the classic serum parameters for liver injury, markers related to the metabolic syndrome might be more valuable to assess damage to the liver in this particular setting. Among possible candidates is adiponectin, an adipose tissue derived adipokine with insulin sensitizing properties, found reduced in obesity. Several study groups elucidated the importance of adiponectin for diabetes^37^,^38^. Adiponectin was shown to increase the insulin-induced tyrosine phosphorylation of the insulin receptor in skeletal muscle as well as to increase whole-body sensitivity to insulin^39^. Hui *et al.* found that hypoadiponectinemia predicts carotid intima media thickness progression, independent of known predictive factors such as age, smoking, hyperlipidemia, and hypertension^40^. Carotid intima thickness is associated with diabetes and NAFLD^41^,^42^. In the present study serum adiponectin concentrations were inversely correlated to BMI and classic parameters of liver damage. Moreover, adiponectin was one of three most important factors for random forests to predict diabetes from serum derived markers. Interestingly performance of logistic regression models to predict diabetes with simple parameters was almost similar for HbA1c + adiponectin and HbA1c + BMI. Addition of both parameters to the model (HbA1c, adiponectin, and BMI) did not further improve the performance. This result suggests, that adiponectin may represent an objective marker for adipocyte function, with similar relevance as BMI. Moreover, the differences between the models HbA1c and HbA1c + adiponectin were also significant at very low FPRs, while this was not true for HbA1c + BMI. Thus the HbA1c + adiponectin model should be preferred in settings where a very high specificity is needed. Generally BMI can be determined easier, faster and cheaper than adiponectin. Though, there are cases (i.e. in highly trained athletes, very small or very large individuals), where BMI is not a reliable estimate for body composition (or adipocyte function)^43^,^44^, although it is a valid estimate in most situations. This might explain the slightly better performance of adiponectin, which needs confirmation in larger studies. Collection of adiponectin data in large cohorts may also enhance our understanding of this marker for adipocyte function and mechanisms leading from obesity to insulin resistance and metabolic syndrome.

Another factor associated with metabolic syndrome is vitamin D, which was found reduced in obesity and has been linked to type 1 and 2 diabetes^45^,^46^,^47^. Histological and clinical stages of NAFLD have also been associated with VD levels in several studies^48^. However, these observations could not be confirmed as causal relationship in some studies^49^,^50^. Among other factors this might be due to the highly complex interaction of the VD and the TGF-β pathway^51^. In particular polymorphisms in the VD receptor gene might impact the influence of VD on liver disease progression^52^. Additionally VD supplementation has been shown to affect adipocytokine levels^53^. This effect might contribute indirectly to the association of low VD with adverse metabolic profiles. In the HNR cohort we were able to show an association between VD levels and serum ALT and AST. VD was also among possible relevant predictors of diabetes. Taken together serum markers related to metabolic syndrome, as adiponectin or VD, can predict prevalence of diabetes in a population based cohort. Moreover, these parameters might also represent candidates for non-invasive markers of NAFLD or NASH. Due to the correlation to classic liver damage markers, even within normal ranges, and the connection to the metabolic syndrome further studies are warranted to elucidate the potential of adipokines and VD to assess metabolic liver injury.

In summary a strong association between transaminase levels, BMI, adiponectin, VD, and diabetes was found in the HNR study. This association was present despite transaminase concentrations within normal range. Adiponectin and HbA1c can predict diabetes with high accuracy in this population based cohort. Re-assessment of current normal range limits should be considered for classic liver transaminases to provide an improved focus concerning chronic metabolic liver injury. Adipokines or other markers related to the metabolic syndrome should be evaluated as possible NAFLD-specific liver injury markers, especially in individuals at risk for metabolic syndrome.

## Patients and Methods

### Study population

The Heinz Nixdorf Recall (HNR) Study is an ongoing population-based prospective cardiovascular cohort study of the Ruhr area in Germany. Random samples of the general population were drawn from residents’ registration offices including both genders aged 45–75 years. People were invited by mail (one invitational letter plus a maximum of two reminder letters) and phone calls to participate. Most people decided to participate after one invitational letter (52.6%)^54^. Blood tests were performed in fasting state, risk factors for coronary artery disease were analyzed by standardized questionnaires. A detailed description of the study design and population has been published previously^55^. Informed consent was obtained from the included participants. The study protocol conformed to the ethical guidelines of the 1975 Declaration of Helsinki and was approved by the institution’s human research committee (Ethikkommission am Universitätsklinikum Essen).

Diabetes mellitus was defined as a prior physician diagnosis of diabetes or taking an anti-diabetic drug. Unknown diabetes was considered when (1) fasting glucose was ≥7.0 mmol/L (60% of study participants with fasting status) or random blood glucose ≥11.1 mmol/L (remaining subjects with less than 8 hours non-fasting status) and (2) subjects had not reported a diagnosed diabetes or antidiabetic medication. For the purpose of the present study known diabetes and unknown diabetes were grouped together as “diabetes”.

### Statistical data analysis and mathematic models

For correlation analyses Pearson product-moment correlation r, point-biserial correlation coefficient r_pb_, and the phi coefficient ϕ were employed, depending on the type of parameters used (quantitative or dichotomous).

Random forests (RFs) we used to identify the most important parameters for the prediction of diabetes. We used the RF implementation in the R package randomForest (http://www.r-project.org/). Earlier studies have shown that RFs are excellent non-linear classifiers, which are highly stable and robust in comparison to other classifiers^19^,^20^. Additionally, RFs provide an importance analysis, which can be used to identify the most important positions for the classification process. The theoretical complexity of the random forest is Θ(MKÑlog^2^Ñ), which is based on the complexity of building single trees (Θ(KNlog^2^N)), with K: the number of variables at each node, N: the number of samples. Due to the fact that the random forests use bootstrap replicates, N is reduced to Ñ = 0.632N. However, a random forest uses M randomized trees (bagging). In the current study, we used the random forests only for variable importance measurement, thus they have only been calculated once and are not used for prediction. For the importance analysis in our dataset the random forest needed 4 seconds on an Intel-Core i7-4700MQ CPU @ 2.40 Ghz. To reduce the bias due to the class imbalance in the dataset, we repeated sub-sampling for 100 times^56^. The most important parameters were then used to build logistic regression models to identify patients with diabetes (as defined above) within the analyzed cohort. For evaluation of the models, we calculated the Receiver Operating Characteristics (ROC) and the corresponding Area Under the Curve (AUC) from a leave-one-out cross-validation. For comparisons between the different models we used the method of De Long *et al.* on the AUC values as well as McNemar’s tests at certain false-positive rates (FPR), namely 0.01, 0.05, and 0.1.

## Additional Information

**How to cite this article**: Kälsch, J. *et al.* Normal liver enzymes are correlated with severity of metabolic syndrome in a large population based cohort. *Sci. Rep.* **5**, 13058; doi: 10.1038/srep13058 (2015).

## Supplementary Material

Supplementary Table 1

## Figures and Tables

**Table 1:** Demographic data of the analyzed study cohort (Heinz Nixdorf Recall).

	Male (n = 2395)	Female (n = 2419)
age (y)	59.7 ± 7.8	59.6 ± 7.8
height (cm)	174.8 ± 6.8	162.1 ± 6.2***
weight (kg)	86.2 ± 13.2	72.6 ± 13.8***
BMI (kg/m^2^)	28.2 ± 4.0	27.7 ± 5.2***
waist circumference (cm)	100.3 ± 10.8	88.5 ± 12.9***
Diabetes n (%)	418 (17.5)	238 (9.8)***
smoking status		
current	614 (25.6%)	514 (21.3%)
former	1105 (46.1%)	557 (23.0%)
never	669 (27.9%)	1345 (55.6%)

***p < 0.0001 *vs.* males.

**Table 2:** Correlations of classic liver serum markers and metabolic parameters in the Heinz Nixdorf Recall study population.

Parameters		r	p
BMI	Adiponectin	−0.2195	<0.0001
BMI	Vitamin D	−0.1243	<0.0001
BMI	ALT/GPT	0.2399	<0.0001
BMI	AST/GOT	0.127	<0.0001
BMI	GGT	0.1221	<0.0001
HbA1c	ALT/GPT	0.1237	<0.0001
HbA1c	AST/GOT	0.0448	<0.01
HbA1c	GGT	0.0907	<0.0001
HbA1c	Adiponectin	−0.1456	<0.0001
HbA1c	Vitamin D	−0.0678	<0.0001
GGT	CRP	0.0798	<0.0001
Diabetes	ALT/GPT	0.1394 (rpb)	<0.0001
Diabetes	AST/GOT	0.0973	<0.0001
Diabetes	GGT	0.1362	<0.0001

**Table 3:** Importance of parameters for diabetes prediction from the Heinz Nixdorf Recall cohort.

Parameter	Mean decrease of Gini impurity*	SD^4^
HbA1c	189.69	5.01
Adiponectin	67.72	3.43
BMI^1^	63.49	3.11
GGT^2^	55.47	2.70
Vitamin D	50.98	1.63
CRP^3^	46.43	1.65
ALT	41.43	1.42
AST	34.07	1.40

^*^The Gini impurity gives an estimate, which parameters are most important for the random forest to predict the condition of interest (in this case: diabetes). A higher decrease of the Gini impurity represents a higher importance for this parameter. 1: Body mass index; 2: gamma-Glutamyltranerase; 3:C-reactive protein; 4: standard deviation.
