# Supplementary material for: Normal liver enzymes are correlated with severity of metabolic syndrome in a large population based cohort
Source: Sci Rep. 2015 Aug 13;5:13058. doi: 10.1038/srep13058 (PMC4535035; doi:10.1038/srep13058)
Supplement: Supplementary Table 1 [file srep13058-s1.doc]

**Normal liver enzymes are correlated with severity of metabolic syndrome in a large population based cohort**

Julia Kälsch, Lars P Bechmann, Dominik Heider, Jan Best, Paul Manka, Hagen Kälsch, Jan-Peter Sowa, Susanne Moebus, Uta Slomiany, Karl-Heinz Jöckel, Raimund Erbel, Guido Gerken and Ali Canbay

**Supplementary Table 1. Metabolic profile of individuals with ALT in normal ranges vs. individuals with elevated ALT**.

|  | ALT^1^ in norm  (n=4708) | ALT elevated  (n=84) | P value |
| --- | --- | --- | --- |
| age (y) | 59.7 ± 7.8 | 58.5 ± 7.7 | 0.17 |
| BMI^2^ (kg/m²) | 27.9 ± 4.6 | 30.8 ± 5.1 | **< 0.0001** |
| waist circumference (cm) | 94.3 ± 13.2 | 101.8 ± 14.0 | **< 0.0001** |
| Cardiac disease n (%) | 321 (6.8) | 4 (4.8) | 0.75 |
| Diabetes n (%) | 631 (13.4) | 23 (27.4) | **0.026** |
| HbA1c (%) | 5.5 ± 0.85 | 5.7 ± 1.2 | 0.13 |
| Fasting Glucose (mg/dl) | 111.5 ± 28.2 | 118.7 ± 34.4 | 0.058 |
| LDL^3^ (mg/dl) | 145.3 ± 36.1 | 153.6 ± 41.5 | **0.038** |
| HDL^4^ (mg/dl) | 58.0 ± 17.2 | 55.9 ± 17.4 | 0.26 |
| Adiponectin (µg/ml) | 9.5 ± 5.9 | 7.9 ± 4.3 | **0.001** |
| Vitamin D (ng/ml) | 16.1 ± 8.3 | 13.4 ± 6.3 | **0.0007** |
| AST^5^ (U/l) | 12.7 ± 3.5 | 31.0 ± 13.4 | **< 0.0001** |
| GGT^6^ (U/l) | 20.4 ± 23.2 | 94.0 ± 134.0 | **< 0.0001** |

1: alanine aminotransferase (normal range for women < 35U/l, for men < 50U/l); 2: Body mass index; 2: low density lipoprotein; 3: high density lipoprotein; 4: aspartate aminotransferase; 5: gamma-Glutamyltranferase.
